# Energy Storage Overflow-Aware Data Delivery Scheme for Energy Harvesting Wireless Sensor Networks

**DOI:** 10.3390/s19061383

**Published:** 2019-03-20

**Authors:** Wenwei Lu, Yi-hua Zhu, Kaikai Chi

**Affiliations:** 1 School of Computer Science & Technology, Zhejiang University of Technology, Hangzhou 310023, China; lww@zafu.edu.cn (W.L.); kkchi@zjut.edu.cn (K.C.); 2 School of Information Engineering, Zhejiang Agriculture and Forestry University, Hangzhou 311300, China

**Keywords:** energy harvesting wireless sensor network, data delivering, energy conservation, energy storage overflow

## Abstract

In Energy-Harvesting Wireless Sensor Networks (EH-WSNs), energy storage with limited capacity is used in the nodes to store the harvested energy. Energy storage overflow (ESO) happens when the energy storage is full, which causes the nodes to be unable to store the newly harvested energy. In traditional data delivery schemes, there is the problem of “energy hungry and surplus coexistence”, meaning that some nodes in the network are hungry for energy while some other nodes continue to waste energy due to ESO. To alleviate this problem, in this paper, we present the ESO-aware multiple path (EAMP) data delivery scheme so that more data can be delivered to the sink. With the EAMP, multiple disjoint paths from the source node to the sink are constructed, and the source node splits data into multiple pieces with each going through one of the paths, which helps in mitigating ESO. Simulation results show that the proposed EAMP scheme can deliver more data than the existing ones.

## 1. Introduction

In traditional battery-powered wireless sensor networks (WSNs), it is inevitable to replace batteries in the nodes when the battery energy no longer supports the nodes’ normal operation. The inconvenience of replacing batteries can be overcome by energy harvesting WSN (EH-WSN) or battery-free WSN (BF-WSN). In the EH-WSN, the nodes do not use batteries, rather they harvest energy from ambient energy sources, such as sunlight, wind, radio, vibration, etc. [1].

As the key task in the EH-WSN, the data delivery scheme needs at least one path from the source node to the destination node before the data can be delivered. The popular metrics applied in building the data delivery path(s) in the EH-WSN include energy harvest rate, energy consumption rate, and residual energy of the nodes, in addition to the common ones, such as packet delay, number of hops, data load, and more. Usually, energy storage is used in the EH-WSN nodes to store the harvested energy [2,3]. Unfortunately, the existing data delivery schemes do not consider the energy missed by the nodes with full energy storage.

Clearly, when an energy storage is full, the newly harvested energy cannot be stored anymore, which is referred to as Energy Storage Overflow (ESO). ESO is ignored in the existing data delivering schemes for EH-WSNs because these schemes usually focus on delivering data to the sink with minimum energy consumption, which may lead to the problem of “energy hungry and surplus coexistence” in the EH-WSN. That is, some nodes are hungry for energy, which may interrupt data delivery when the energy-hungry nodes exhaust their energy, whereas other nodes continuously experience ESO. In fact, the data delivery process can last longer without interruption so that more data can be delivered if we consider ESO in the data delivery scheme. Motivated by this, we study the ESO-aware data delivery scheme. The main contributions of the paper are as follows:

(1) We propose the ESO-aware multiple path (EAMP) data delivery scheme, in which multiple disjoint paths from the source node to the sink are constructed to deliver data. With the EAMP, the source node splits data into multiple pieces with each going through one of the paths, which mitigates ESO and avoids the problem of “energy hungry and surplus coexistence”.

(2) For the EAMP, we formulate an optimization problem that simultaneously minimizes the total ESO and maximizes the total residual energy. Then, we solve the optimal problem by using a genetic algorithm, which leads to the optimal Data Delivering Decision (DDD) applied in the EAMP.

(3) Simulation results show that the proposed scheme can deliver more data than the existing schemes in addition to maintaining higher residual energy and lower ESO.

The remainder of the paper is organized as follows. Related works are surveyed in Section 2. The EAMP, the optimization problem for the EAMP, and the genetic algorithm are proposed in Section 3. Simulation results are presented in Section 4. Section 5 concludes the paper.

## 2. Related Work

In EH-WSNs, the nodes scavenge energy from ambient energy sources, such as solar, thermal, wind, vibration [1], or radio frequency [2,3] sources. With advances in energy-harvesting techniques, it becomes feasible to build a sustainable EH-WSN capable of harvesting energy from the environment to support long-term applications [4]. To describe the EH-WSN applications effectively in simulation environment, SensEH [5] is presented to provide a unified framework that fills the gap between simulation and deployment in the EH-WSN.

As in any network, data delivery is the key task in the EH-WSN. An optimal data-gathering algorithm for dynamic sensing and routing (DoSR) was proposed [6] to jointly optimize data sensing and data transmission while guaranteeing fairness for the EH-WSN. The routing algorithm with competitive ratio was proposed in [7], which involves the factors of energy consumption, available energy, and energy harvesting rate. The opportunistic routing mechanism was presented in [8] deals with unpredictable duty cycle and wireless channel unreliability by using wireless broadcast nature. The epidemic routing mechanism was applied in the EH-WSN to address the uncertainty of sensors’ availability and dynamic network topology [9]. A mobile collector was applied to collect data from designated sensors and balance energy consumption in a solar-powered network [10]. A mobile sink was adopted to improve data collection rate [11]. A fair and high throughput data collection scheme was proposed for the EH-WSN [12], which computes the optimal lexicographic rate assignment under the constraint of no node running out of energy. To support multihop data gathering in the EH-WSN, relay node placement algorithms were proposed [13], which consider network connectivity and survivability. A coding scheme was applied in gathering data for a multihop EH-WSN [14]. The energy-harvesting-aware routing algorithm (EHARA) [15] is proposed to improve the lifetime of sensor nodes as well as the quality-of-service (QoS) under variable energy availability conditions. The online algorithm called optimal scheduling algorithm (OSCAR) proposed in [16] makes decisions on system state, energy harvesting, and data transmission with mobile energy harvested sensing devices so as to achieve close-to-optimal transmission utility performance. The FDEHSN protocol presented in [17] considered the application that the source forwards data to the destination via an energy-harvesting two-antenna relay node. The scenario of an energy-harvesting transmitter sending messages to two users over parallel and fading Gaussian broadcast channels was studied in [18]. As a viable solution to saving energy, compressive sensing is used in [19], which can improve the lifetime of the device in WSNs. The above surveyed data schemes share the same shortcoming that they do not take ESO into account. There are a few data delivery schemes that consider ESO. Route selection schemes that consider network energy wastage due to overcharge of finite-capacity batteries were proposed in [20] and [21], in which the cost associated with the packet transmission energy consumption and the energy wastage due to battery overcharge is minimized [20] and the wastage due to overcharging of a finite battery is predicted [21]. Although these schemes take ESO into account, they exhibit the problem of uneven energy consumption because they let the source node deliver all data to the destination over a predetermined route. This causes the nodes participating in data delivery to consume much more energy than those not participating in data delivery. The uneven energy consumption problem is considerably alleviated in the proposed EAMP because multiple disjoint paths are used in delivering packets, which differs from the existing ESO-aware data delivery schemes.

## 3. ESO-Aware Data Delivering Scheme

In this section, we present the ESO-aware multiple paths (EAMP) data delivery scheme, which uses multiple disjointed paths. In the following, we use the terms “path” and “route” interchangeably.

First, we present an example to show the main idea underlying the proposed EAMP.

### 3.1. An Example of Alleviating ESO

**Figure 1:**
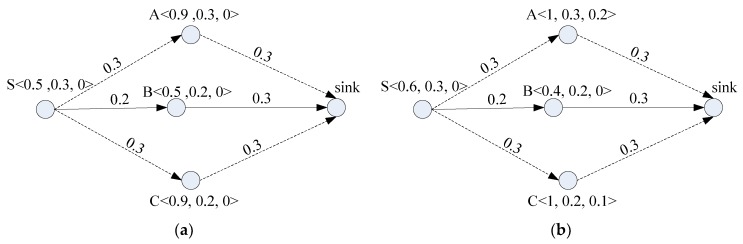
Traditional routing. (**a**) Before data delivery; (**b**) After data delivery using route S→B→Sink.

In Figure 1, each node’s energy storage capacity is unified to 1. The notation “<*a*, *b*, *c*>” beside a node means that: the node’s current residual energy is *a*, the incoming energy (i.e., the predicted energy to be harvested) is *b*, and ESO is c. For instance, notation “S <0.5, 0.3, 0>” in Figure 1a means that node S is with residual energy of 0.5 unit of energy (UoE), incoming energy of 0.3 UoE, and ESO of 0 UoE. In addition, an arrow in the figure stands for a wireless link, and the number beside the arrow stands for the energy consumption for delivering one unit of data. In Figure 1a, delivering one unit of data over wireless links of S→A, S→B, and S→C expends 0.6, 0.5, and 0.6 UoE, respectively.

A routing protocol with the goal of saving energy usually chooses the route with the minimum energy consumption. As a result, it chooses the minimal energy consumption route S→B→sink to deliver data, which expends 0.5 UoE per unit of data. After one unit of data is delivered to the sink via this route, variation in the residual energy and ESO of all the nodes is shown in Figure 1b. This figure indicates that nodes A and C are fully charged, i.e., their residual energy is 1 UoE, and node A produces ESO of 0.2 UoE because only 0.1 UoE out of the incoming energy (0.3 UoE) can be stored while node C produces ESO of 0.1 UoE. In addition, node B consumes 0.3 UoE but harvests 0.2 UoE, causing its residual energy to be 0.4 UoE. Similarly, node S has residual energy of 0.6 UoE. Thus, the total residual energy in the BF-WSN is 3 UoE.

**Figure 2:**
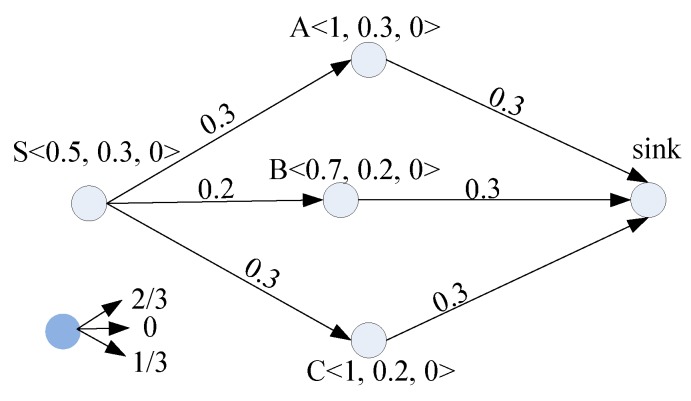
Energy storage overflow (ESO)-reducing routing.

In fact, the total residual energy and ESO can be improved if we let node S split data into multiple pieces and then deliver these pieces over multiple routes to the sink. This idea is illustrated in Figure 2, where node S breaks one unit of data into two pieces as 2/3 and 1/3 of the data, respectively, and the two pieces go through routes S→A→sink and S→C→sink (the two routes contain the nodes with incoming energy), respectively. Thus, links of S→A and A→sink in the upper route expend 0.2 UoE each since only 2/3 units of data are delivered, while each of the links in the lower route consumes 0.1 UoE due to only 1/3 units of data being delivered. Hence, after the data are simultaneously delivered to the sink through the two routes, the residual energy of node S remains unchanged since the incoming energy (0.3 UoE) can be consumed in delivering two portions of the one unit of data that go through two separated routes. Additionally, node A consumes 0.2 UoE out of the incoming energy, and the left 0.1 UoE is stored, which makes its residual energy 1 UoE. The residual energy of other nodes can be similarly obtained. It should be pointed out that, with this scheme, no ESO is introduced and the total residual energy is 3.2 UoE. That is, both ESO and total residual energy in this scheme are better than the previous one.

In summary, ESO can be reduced and the total residual energy can be increased by delivering data over multiple routes, which helps in extending network lifetime and delivering more data. This is the main idea underlying our EAMP.

### 3.2. The EAMP

**Figure 3:**
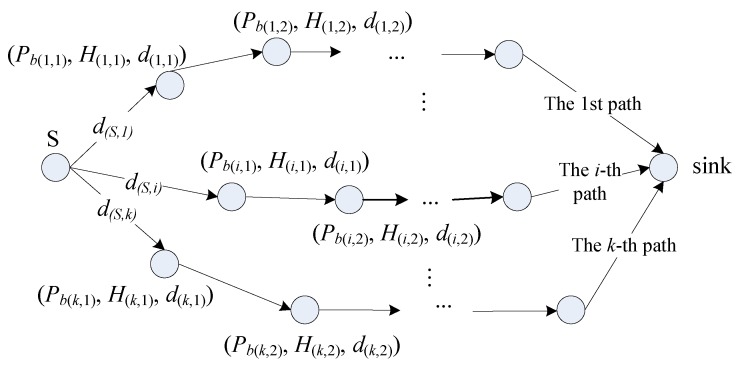
Packet delivery over *k* disjointed paths.

Assume the EH-WSN has one sink and *N* sensor nodes. Each node harvests energy from an ambient energy source and the harvested energy is stored in its energy storage with capacity of *B* (in Joule). Assume the source node *S* has *k* disjoint paths to the sink. We use *R_i_* to represent the set of the nodes on the *i*-th path, exclusive of *S*, and |*R_i_*| stands for the number of the elements in *R_i_*. In addition, notation “(*i*, *j*)” represents the *j*-th node on the *i*-th path. Thus, *R_i_* = {(*i*,1), (*i*,2), …, (*i*,|*R_i_*|)}, *i* = 1, 2, …, *k*. Moreover, for node (*i*, *j*), *P_b_*_(*i*,*j*)_ and *H*_(*i*,*j*)_ stands for its residual energy before data delivery and incoming energy during the period of data delivery, respectively, and *d*_(*i*,*j*)_ denotes the distance between nodes (*i*, *j*) and (*i*, *j* + 1). *d*_(*S*,*i*)_ represents the distance between the source node *S* and node (*i*,1) (*i* = 1, 2, …, *k*). The scenario with *k* disjoint paths is shown in Figure 3 and the notations used are summarized in Table 1.

Let *u* be the minimum Transmission Unit (TU) in bits. Assume node *S* intends to deliver *L* TUs of data, i.e., *Lu* bits, to the sink over the *k* paths. We let the source node apply Data Delivering Decision (DDD), denoted by vector φ = ($\varphi_{1}$, $\varphi_{2}$, …, $\varphi_{k}$), in which $\varphi_{i}$ ≥ 0 stands for the number of TUs to be delivered over the *i*-th path. Thus, we have

(1) $$\sum_{i=1}^{k}\varphi_{i}=L.$$

Before moving further, we derive the energy consumption based on the energy model presented in [22]. That is, the energy consumed in transmitting *u* bits to the receiver with *d* meters away is

(2) $$E_{Tx}(u,d)=u(\varepsilon_{0}+\varepsilon_{1}d^{\gamma }),$$

and the energy consumed in receiving *u* bits is

(3) $$E_{Rx}(u)=u\varepsilon_{0},$$

here, $\varepsilon_{0}$ denotes the energy consumption resulting from digital coding, modulation, filtering, and spreading of the signal; $\varepsilon_{1}$ is the energy consumed by the transmitter power amplifier; and *γ* is path loss parameter valued in 2 through 4 [22]. As a result, under the DDD *φ*, the energy expended by node (*i*, *j*) in forwarding data packets, which contains the energy consumed for receiving the packets from its preceding node and the energy consumed for transmitting to the next node, is

(4) $$\begin{array}{l} E_{\left(i,j\right)}(\varphi )=E_{Rx}(\varphi_{i}u)+E_{Tx}(\varphi_{i}u,d_{\left(i,j\right)})\\ \;=\varphi_{i}u[2\varepsilon_{0}+\varepsilon_{1}(d_{\left(i,j\right)}{)}^{\gamma }],i=1,2,\cdots ,k,j=1,2,\cdots ,|R_{i}|. \end{array}$$

Additionally, the energy expended by the source node *S* for delivering the data with size of $\varphi_{i}u$ to node (*i*, 1) is as follows:

(5) $$E_{i}(\varphi )=E_{Tx}(\varphi_{i}u,d_{\left(S,i\right)})=\varphi_{i}u[\varepsilon_{0}+\varepsilon_{1}{\left(d_{\left(S,i\right)}\right)}^{\gamma }],i=1,2,\cdots ,k.$$

Upon completion of the DDD *φ*, the ESO at node (*i*, *j*) is

(6) $${\hat{E}}_{\left(i,j\right)}={\left[P_{b(i,j)}+H_{\left(i,j\right)}-E_{\left(i,j\right)}(\varphi )-B\right]}^{+},$$

where *B* is the energy storage capacity and notation “[.]^+^” is a rectifier function defined by

(7) $${\left[x\right]}^{+}=\left\{ \begin{array}{l} x,x\geq 0;\\ 0,x<0. \end{array}\right.$$

Thus, the residual energy of node (*i*, *j*) after data delivery is

(8) $$P_{a(i,j)}=P_{b(i,j)}+H_{\left(i,j\right)}-E_{\left(i,j\right)}(\varphi )-{\hat{E}}_{\left(i,j\right)}.$$

Similarly, after completion of the DDD *φ*, the ESO at node *S* is

(9) $${\hat{E}}_{\left(S\right)}={\left[P_{b(S)}+H_{\left(S\right)}-\sum_{i=1}^{k}E_{i}(\varphi )-B\right]}^{+},$$

where *P_b_*_(*S*)_ and *H*_(*S*)_ represent the residual energy of node *S* before data delivery and the incoming energy during the period of data transmission at node *S*, respectively. The residual energy of node *S* after data delivery is

(10) $$P_{a(S)}=P_{b(S)}+H_{\left(S\right)}-\sum_{i=1}^{k}E_{i}(\varphi )-{\hat{E}}_{\left(s\right)}.$$

It is required that a node is permitted to transmit packets only when its residual energy after data delivery is positive, i.e.,

(11) $$P_{a(i,j)}>0,$$

and

(12) $$P_{a(S)}>0,$$

which leads to the prerequisite condition of successful forwarding data packets from node (*i*, *j*) to the sink under the DDD *φ* as follows:

(13) $$P_{b(i,j)}+H_{\left(i,j\right)}-E_{\left(i,j\right)}(\varphi )>{\hat{E}}_{\left(i,j\right)},$$

and meanwhile the source node *S* is required to satisfy

(14) $$P_{b(S)}+H_{\left(S\right)}-\sum_{i=1}^{k}E_{i}(\varphi )>{\hat{E}}_{\left(s\right)}.$$

Substituting (4), (5), (6), and (9) into (8) and (10), we obtain

(15) $$\left\{ \begin{array}{l} P_{b(S)}+H_{\left(S\right)}-u\sum_{i=1}^{k}\varphi_{i}[\varepsilon_{0}+\varepsilon_{1}{\left(d_{\left(S,i\right)}\right)}^{\gamma }]>{\left[P_{b(i,j)}+H_{\left(i,j\right)}-E_{\left(i,j\right)}(\varphi )-B\right]}^{+};\\ P_{b(i,j)}+H_{\left(i,j\right)}-\varphi_{i}u[2\varepsilon_{0}+\varepsilon_{1}(d_{\left(i,j\right)}{)}^{\gamma }]>{\left[P_{b(S)}+H_{\left(S\right)}-\sum_{i=1}^{k}E_{i}(\varphi )-B\right]}^{+}. \end{array}\right.$$

From (4), (5), (8), and (10), we obtain the total residual energy after data delivery of all the nodes taking part in forwarding the data packets under the DDD *φ* as follows:

(16) $$\begin{array}{l} P_{tot}(\varphi )=P_{a(S)}+\sum_{i=1}^{k}\sum_{j=1}^{\left|R_{i}\right|}P_{a(i,j)}\\ =P_{b(S)}+H_{\left(S\right)}-\sum_{i=1}^{k}E_{i}(\varphi )-{\hat{E}}_{\left(S\right)}+\sum_{i=1}^{k}\sum_{j=1}^{\left|R_{i}\right|}[P_{b(i,j)}+H_{\left(i,j\right)}-E_{\left(i,j\right)}(\varphi )-{\hat{E}}_{\left(i,j\right)}]\\ =P_{b(S)}+H_{\left(S\right)}+\sum_{i=1}^{k}\sum_{j=1}^{\left|R_{i}\right|}[P_{b(i,j)}+H_{\left(i,j\right)}]-\{\sum_{i=1}^{k}\varphi_{i}u[\varepsilon_{0}+\varepsilon_{1}{\left(d_{\left(S,i\right)}\right)}^{\gamma }]+\sum_{i=1}^{k}\sum_{j=1}^{\left|R_{i}\right|}\varphi_{i}u[2\varepsilon_{0}+\varepsilon_{1}{\left(d_{\left(i,j\right)}\right)}^{\gamma }]\}\\ \begin{array}{cc} & -\{{\left[P_{b(S)}+H_{\left(S\right)}-B-\sum_{i=1}^{k}\varphi_{i}u[\varepsilon_{0}+\varepsilon_{1}{\left(d_{\left(S,i\right)}\right)}^{\gamma }]\right]}^{+}+\sum_{i=1}^{k}\sum_{j=1}^{\left|R_{i}\right|}{\left[P_{b(i,j)}+H_{\left(i,j\right)}-B-\varphi_{i}u[2\varepsilon_{0}+\varepsilon_{1}{\left(d_{\left(i,j\right)}\right)}^{\gamma }]\right]}^{+}\} \end{array}. \end{array}$$

Additionally, from (4), (5), (6), and (9), we obtain the total ESO in the nodes under the DDD *φ* as follows:

(17) $$\begin{array}{l} {\hat{E}}_{tot}(\varphi )={\hat{E}}_{\left(S\right)}+\sum_{i=1}^{k}\sum_{j=1}^{\left|R_{i}\right|}{\hat{E}}_{\left(i,j\right)}\\ ={\left[P_{b(S)}+H_{\left(S\right)}-\sum_{i=1}^{k}E_{i}(\varphi )-B\right]}^{+}+\sum_{i=1}^{k}\sum_{j=1}^{\left|R_{i}\right|}{\left[P_{b(i,j)}+H_{\left(i,j\right)}-E_{\left(i,j\right)}(\varphi )-B\right]}^{+}\\ ={\left[P_{b(S)}+H_{\left(S\right)}-B-\sum_{i=1}^{k}\varphi_{i}u[\varepsilon_{0}+\varepsilon_{1}{\left(d_{\left(S,i\right)}\right)}^{\gamma }]\right]}^{+}+\sum_{i=1}^{k}\sum_{j=1}^{\left|R_{i}\right|}{\left[P_{b(i,j)}+H_{\left(i,j\right)}-B-\varphi_{i}u[2\varepsilon_{0}+\varepsilon_{1}{\left(d_{\left(i,j\right)}\right)}^{\gamma }]\right]}^{+}. \end{array}$$

Taking (16) and (17), we now formulate the Optimization Problem (OP) in (18) that minimizes the total ESO and maximizes the total residual energy simultaneously, in which Equations (1) and (15) are used as constraints.

(18) $$\begin{array}{l} Min\;\alpha {\hat{E}}_{tot}(\varphi )+(1-\alpha )[P_{tot}(\varphi ){]}^{-1}\\ w.r.t.\;\varphi_{1},\varphi_{2},\cdots ,\varphi_{k}\\ s.t.\;\left\{ \begin{array}{l} \sum_{i=1}^{k}\varphi_{i}=L;\\ P_{b(S)}+H_{\left(S\right)}-u\sum_{i=1}^{k}\varphi_{i}[\varepsilon_{0}+\varepsilon_{1}{\left(d_{\left(S,i\right)}\right)}^{\gamma }]>{\left[P_{b(i,j)}+H_{\left(i,j\right)}-E_{\left(i,j\right)}(\varphi )-B\right]}^{+};\\ P_{b(i,j)}+H_{\left(i,j\right)}-\varphi_{i}u[2\varepsilon_{0}+\varepsilon_{1}(d_{\left(i,j\right)}{)}^{\gamma }]>{\left[P_{b(S)}+H_{\left(S\right)}-\sum_{i=1}^{k}E_{i}(\varphi )-B\right]}^{+}, \end{array}\right. \end{array}$$

where *α* is a real number valued in [0,1] and it is used to balance ESO and residual energy. We set *α* to a constant close to 1 if emphasize ESO and close 0 otherwise. Obviously, when *α* = 1, the OP reduces to minimize the total ESO, which will be referred to as the OP-ESO, and when *α* = 0, the OP just maximizes the total residual energy, which will be referred to as the OP-RE.

### 3.3. Solution of the Optimization Problem

**Figure 4:**
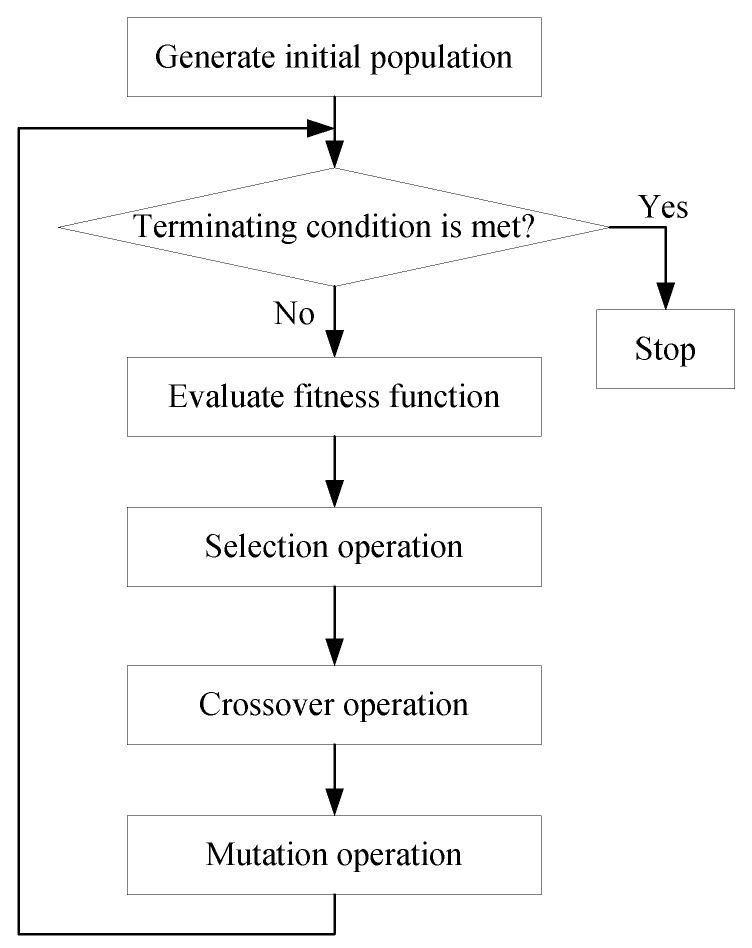
Genetic algorithm (GA).

To solve the OP in (18), although the number of the DDDs that satisfy the first constraint (i.e., Equation (1)) is finite, it is hard to enumerate and check each of these DDDs to find the solution of the OP. Hence, we resort to genetic algorithm (GA) [23] thanks to its capability of searching for the optimal solution by parallel means. The main operations contained in the GA are shown in Figure 4. In the GA, we choose a DDD as a chromosome. That is, a chromosome is a *k*-dimension tuple.

As shown in Figure 4, the main mechanisms in the GA include selection, crossover, and mutation operations. For any one of the OPs, we use the following GA to find its solution:

(1) Initial population. A chromosome is called “feasible chromosome” of the OP if it satisfies the three constraints in the OP. We set the initial population by randomly generating *M* feasible chromosomes with each being produced by the “feasible chromosome generating process” as follows. To generate a feasible chromosome $\varphi^{\left(j\right)}=(\varphi_{1}^{\left(j\right)},\varphi_{2}^{\left(j\right)},\cdots ,\varphi_{k}^{\left(j\right)})$ (*j*∈{1, 2, …, *M*}), where (*i* = 1, 2, …, *k*) is the number of TUs that flows through the *i*-th path, we first generate random numbers $r_{1},r_{2},\cdots ,r_{k}\in [0,1]$ and then use them to produce *k* integers $\varphi_{1}^{\left(j\right)},\varphi_{2}^{\left(j\right)},\cdots ,\varphi_{k}^{\left(j\right)}$ ∈ [0, *L*] as follows:

(19) $$\{\begin{array}{l} \varphi_{i}^{\left(j\right)}=\left\lfloor L\frac{r_{i}}{\sum_{s=1}^{k}r_{s}}\right\rfloor ,i=1,2,\cdots ,k-1;\\ \varphi_{k}^{\left(j\right)}=L-\sum_{v=1}^{k-1}\varphi_{v}^{\left(j\right)}, \end{array}$$

where ⌊⋅⌋ is the floor function. Obviously, the generated chromosome $\left(\varphi_{1}^{\left(j\right)},\varphi_{2}^{\left(j\right)},\cdots ,\varphi_{k}^{\left(j\right)}\right)$ satisfies (1). If it satisfies the other two constraints in the OP, then it is a feasible chromosome. Otherwise, we regenerate a chromosome in (19) until the one satisfying the other two constraints is produced, which finishes generating the feasible chromosome.

(2) Selection operation. We choose the fitness functions of the OP in (18) as $f(x)=\alpha {\hat{E}}_{tot}(\varphi )+(1-\alpha )\left[P_{tot}(\varphi \right){]}^{-1}$. In the current population, we evaluate each chromosome using its fitness function and select the chromosome with the minimum fitness to be passed to the next generation. In addition, a chromosome is copied into the next generation according to the probability of $f^{-1}(\varphi^{\left(j\right)})/\sum_{j=1}^{M}f^{-1}(\varphi^{\left(j\right)})$ via Roulette wheel (*j* = 1, 2, …, *M*), which indicates that a chromosome with a smaller fitness has more copies in the next generation.

(3) Crossover operation. The chromosomes are randomly paired for crossover. For the pair of $\varphi^{\left(a\right)}=(\varphi_{1}^{\left(a\right)},\varphi_{2}^{\left(a\right)},\cdots ,\varphi_{k}^{\left(a\right)})$ and $\varphi^{\left(b\right)}=(\varphi_{1}^{\left(b\right)},\varphi_{2}^{\left(b\right)},\cdots ,\varphi_{k}^{\left(b\right)})$ to be crossed over, we randomly pick an integer, say *v*, in {1, 2, …, *k* − 1} as a crossover point. Then, the crossover operation yields two chromosomes: $\varphi^{\left(a^{\prime }\right)}=(\varphi_{1}^{\left(a^{\prime }\right)},\varphi_{2}^{\left(a^{\prime }\right)},\cdots ,\varphi_{k}^{\left(a^{\prime }\right)})$ and $\varphi^{\left(b^{\prime }\right)}=(\varphi_{1}^{\left(b^{\prime }\right)},\varphi_{2}^{\left(b^{\prime }\right)},\cdots ,\varphi_{k}^{\left(b^{\prime }\right)})$, in which we set

(20) $$\left\{ \begin{array}{l} \varphi_{i}^{\left(a^{\prime }\right)}=\varphi_{i}^{\left(a\right)},i=1,2,\cdots ,v;\\ \varphi_{i}^{\left(a^{\prime }\right)}=\varphi_{i}^{\left(b\right)},i=v+1,v+2,\cdots ,\delta^{\prime };\\ \varphi_{\delta^{\prime }+1}^{\left(a^{\prime }\right)}=L-\sum_{i=1}^{\delta^{\prime }}\varphi_{i}^{\left(a^{\prime }\right)};\\ \varphi_{i}^{\left(a^{\prime }\right)}=0,i=\delta^{\prime }+2,\delta^{\prime }+3,\cdots ,k, \end{array}\right.$$

and

(21) $$\left\{ \begin{array}{l} \varphi_{i}^{\left(b^{\prime }\right)}=\varphi_{i}^{\left(b\right)},i=1,2,\cdots ,v;\\ \varphi_{i}^{\left(b^{\prime }\right)}=\varphi_{i}^{\left(a\right)},i=v+1,v+2,\cdots ,\delta^{\prime \prime };\\ \varphi_{\delta^{\prime \prime }+1}^{\left(b^{\prime }\right)}=L-\sum_{i=1}^{\delta^{\prime \prime }}\varphi_{i}^{\left(b^{\prime }\right)};\\ \varphi_{i}^{\left(b^{\prime }\right)}=0,i=\delta^{\prime \prime }+2,\delta^{\prime \prime }+3,\cdots ,k, \end{array}\right.$$

where

(22) $$\left\{ \begin{array}{l} \delta^{\prime }=\max\{x|\sum_{i=1}^{v}\varphi_{i}^{\left(a^{\prime }\right)}+\sum_{i=v+1}^{x}\varphi_{i}^{\left(b\right)}\leq L,x\leq k-1\};\\ \delta^{\prime \prime }=\max\{x|\sum_{i=1}^{v}\varphi_{i}^{\left(b^{\prime }\right)}+\sum_{i=v+1}^{x}\varphi_{i}^{\left(a\right)}\leq L,x\leq k-1\}. \end{array}\right.$$

The aim of introducing $\delta^{\prime }$ and $\delta^{\prime \prime }$ in (22) is to make the two generated chromosomes $\varphi^{\left(a^{\prime }\right)}$ in (20) and $\varphi^{\left(b^{\prime }\right)}$ in (21) satisfy the first constraint (i.e., Equation (1)) in OP (18). In fact, the crossover operation tries shifting the genes after the crossover point in the two chromosomes. Unfortunately, the newly generated chromosomes may not satisfy the first constraint if the genes after the crossover point are all shifted. Therefore, one of the new chromosome first, say $\varphi^{\left(a^{\prime }\right)}$, inherits the first *v* genes in $\varphi^{\left(a\right)}$, and then we pick the genes behind the crossover point *v* such that the sum of the picked genes and the ones already in $\varphi^{\left(a^{\prime }\right)}$ does not exceed *L*. Then, we generate a new gene to satisfy that the sum of the genes already in $\varphi^{\left(a^{\prime }\right)}$ is equal to *L* so that the first constraint is met. Then, the remaining genes, if any, are set to 0 so that the constraint is still met.

In the case when any of the chromosomes in (20) and (21) does not satisfy the other two constraints of the OP, the involved chromosome(s) will be replaced with the one(s) generated using the aforementioned feasible chromosome generating process.

(4) Mutation operation. Each chromosome is mutated with a small probability. For a chromosome to be mutated, we pick an integer *v* in {1, 2, …, *k*} randomly. If *v* < *k*, the mutation operation switches $\varphi_{v}^{\left(j\right)}$ and $\varphi_{v+1}^{\left(j\right)}$, which leads to a new chromosome as $\left(\varphi_{1}^{\left(j\right)},\cdots ,\varphi_{v+1}^{\left(j\right)},\varphi_{v}^{\left(j\right)},\cdots ,\varphi_{k}^{\left(j\right)}\right)$. Otherwise, $\varphi_{k-1}^{\left(j\right)}$ and $\varphi_{k}^{\left(j\right)}$ are switched, which yields $\left(\varphi_{1}^{\left(j\right)},\varphi_{2}^{\left(j\right)},\cdots ,\varphi_{k}^{\left(j\right)},\varphi_{k-1}^{\left(j\right)}\right)$. If the newly generated chromosome does not satisfy any of the constraints of the OP, it is replaced with the one generated by using the aforementioned feasible chromosome generating process.

After the GA terminates, the solution of the OP is found. The results shown in Section 4 are obtained from the above GA.

Following [7] and [24], we assume that the incoming energy is known by each sensor node over a finite time horizon, and the sink knows the residual energy and incoming energy of all the nodes through the previous information exchange. The sink is assumed to have sufficient power supply and it stores the information of the multiple disjointed paths. Before the sink intends to collect the data, it finds the optimal DDD by solving the OP in (18) and then delivers the optimal DDD to the source node. The source node delivers its data to the sink through the known multiple paths with the received optimal DDD.

## 4. Performance Evaluation

In this section, we evaluate the proposed EAMP via a simulation program written in MATLAB. The parameter values used in the simulation are shown in Table 2.

### 4.1. Simulation Enviroment

In the simulation, 100 nodes are randomly deployed in a square with size of 200 × 200 m^2^. We randomly choose a pair of nodes as the source node and the sink, respectively. Then, multiple disjointed paths between the two nodes are generated using the SMR protocol presented in [25]. As in our previous study [2], we assume the nodes harvest solar energy and adopt the solar irradiation data for Chicago O’Hare International Airport from the National Solar Radiation Data Base, which was published by The U.S. Department of Energy [26]. We consider periods of 4:00–5:00, 7:00–8:00, and 11:00–12:00, which are referred to as low, medium, and high energy harvesting periods, respectively. The average solar radiation received on a global horizontal surface in the low, medium, and high energy harvesting periods are 5.1, 296, and 623 Wh/m^2^, respectively [26]. Thus, we assume a node can averagely harvest energy with low energy-harvesting rate (LER) of 5.1/400, medium energy-harvesting rate (MER) of 296/400, and high energy-harvesting rate (HER) of 623/400 W. These rates correspond to the energy harvested by a device which captures solar energy with a square of 5 × 5 cm^2^ i.e., 1/400 of 1 m^2^. We performed the simulation with these three energy-harvesting rates.

### 4.2. Simulation Results

**Figure 5:**
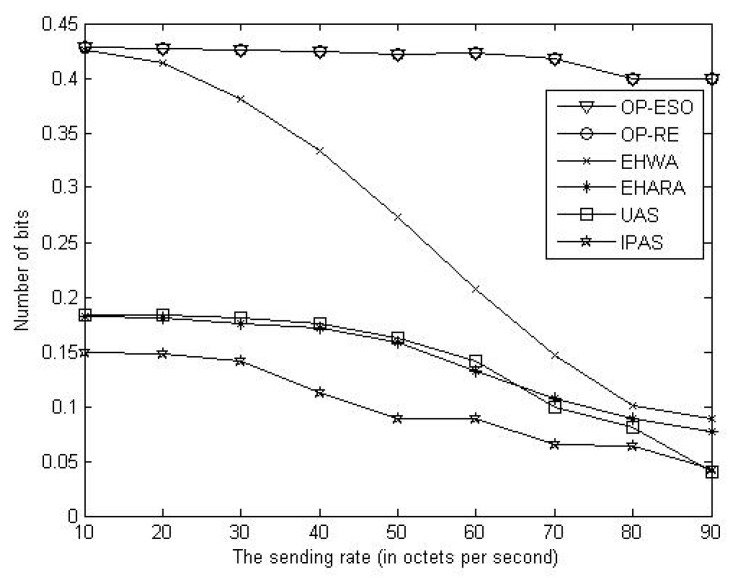
Number of delivered bits vs. data sending rate in the low energy-harvesting rate (LER) case.

**Figure 6:**
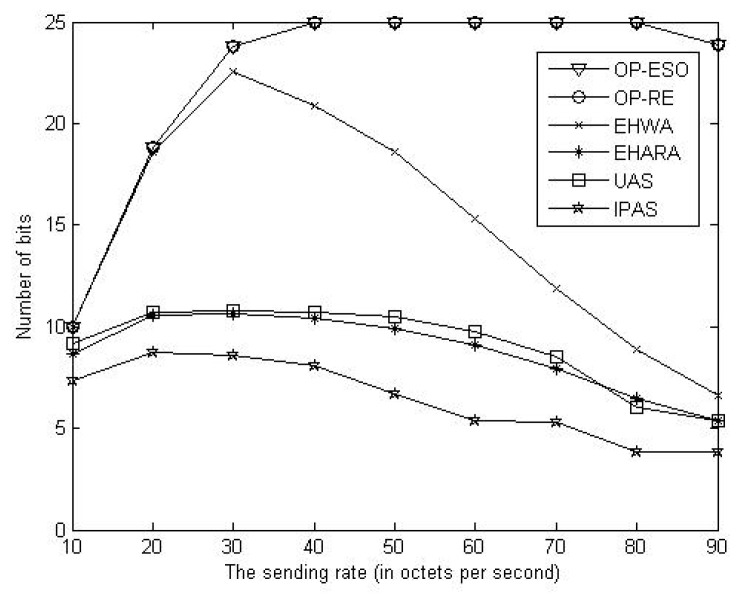
Number of delivered bits vs. data sending rate in medium energy-harvesting rate (MER) case.

**Figure 7:**
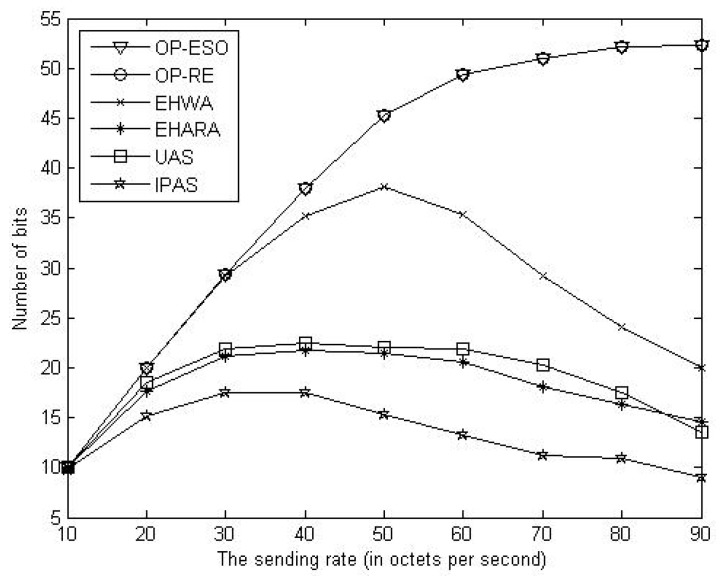
Number of delivered bits vs. data sending rate in high energy-harvesting rate (HER) case.

We compare our OP-ESO and OP-RE with the EHWA [20], the EHARA [15], the uniform allocation scheme (UAS), and inverse proportion allocation scheme (IPAS) in terms of number of bits delivered to the sink, where all paths have the same capacity in the UAS, and each path having capacity inversely proportional to the number of hops in the path in the IPAS. For instance, assume *k* paths have *h*_1_, *h*_2_, …, and *h_k_* numbers of hops. Then, the *i*-th path is allocated to capacity of (1/*h_i_*)/(1/*h*_1_ + 1/*h*_2_ +, …, + 1/*h_k_*) out of the total capacity (*i* = 1, 2, …, *k*). First, we study impact of data sending rate on number of delivered bits. Letting the source send its data for one hour with rates of 10, 20, 30, …, and 90 bytes per second, we obtain Figure 5, Figure 6 and Figure 7. From the figures, we observe that the proposed OP-ESO and OP-RE outperform the other four schemes in number of delivered bits in the LER, MER, and HER cases. Figure 5 indicates that in the LER case, the number of delivered bits reduces as data sending rate grows. The reason is that a higher data sending rate causes more energy to be consumed in delivering data and thus the node needs to spend more time on harvesting energy, which reduces the number of delivered bits. This phenomenon can be alleviated when energy harvesting rate increases, which is reflected in Figure 6 that illustrates the results of the MER case. From Figure 6, when the sending data rate is no more than 80, a higher data sending rate makes the proposed OP-ESO and OP-RE bring in a higher number of delivered bits. The other four schemes present a similar up-then-down trend, i.e., number of delivered bits gradually grows and then goes down. This trend can be clearly seen from Figure 7, in which the HER case is considered. In summary, from Figure 5, Figure 6 and Figure 7, we observe that for a given energy-harvesting rate, the numbers of delivered bits in all the schemes exhibit the up-then-down trend when data sending rates increases.

**Figure 8:**
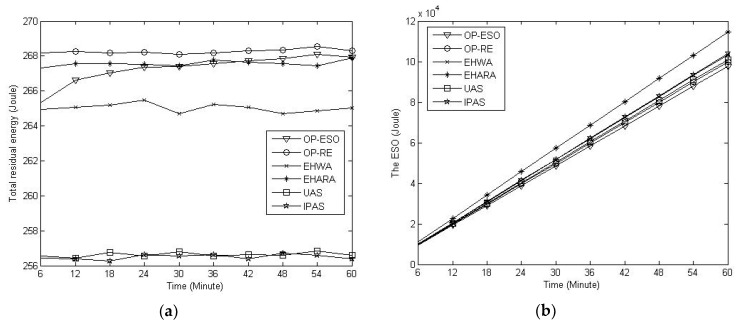
Comparison of residual energy and ESO. (**a**) Total residual energy; (**b**) ESO.

Next, we compare them in terms of residual energy and ESO. We only consider the MER case with a data sending rate of 20 octets per second, which leads to Figure 8. This figure indicates that the OP-RE achieves the maximal residual energy and the OP-ESO achieves the minimal ESO, respectively. That is, the objects of the OP-ESO (i.e., minimizing ESO) and the OP-RE (i.e., maximizing total residual energy) have been realized. Another observation is that the total residual energy fluctuates with time (see Figure 8a) and ESO increases with time (see Figure 8b).

**Figure 9:**
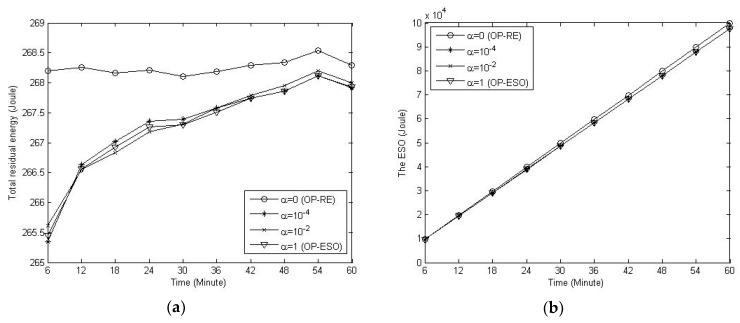
The ESO-aware multiple path (EAMP) scheme with variation in *α*. (**a**) Impact of *α* on residual energy; (**b**) Impact of *α* on ESO.

Last, we study the impact of *α* on residual energy and ESO in the proposed EAMP. Setting *α* = 0 (i.e., the OP-RE), 10^−4^, 10^−2^, and 1 (i.e., the OP-ESO), we obtain Figure 9. From the figure, we have the observations that: 1) the smaller the *α*, the higher the residual energy, and the EAMP with *α* = 0 (i.e., the OP-RE) has the highest total residual energy (see Figure 9a), and ESO is slightly affected by variation in *α* (see Figure 9b).

## 5. Conclusions

In EH-WSN, there is the problem of “energy hungry and surplus coexistence”. That is, some nodes suffer from serious energy shortage while some other nodes waste energy duo to ESO. This problem is alleviated by the proposed EAMP data delivery scheme. The EAMP takes ESO into account and can alleviate overflow of energy storage in the nodes so that the EH-WSN nodes can consume energy evenly. With the EAMP, a node can optimally split data into multiple pieces and simultaneously deliver these pieces over multiple paths to mitigate ESO. The EAMP is applicable to EH-WSNs and enables the EH-WSN nodes to deliver data for a longer period without suffering from interruption due to energy shortage.

It should be pointed out that the proposed schemes may not achieve satisfactory performance when they are applied in cases where multiple source nodes simultaneously deliver data to their respective destinations, meanwhile some source-to-destination paths with different sources share some nodes. In the multiple sources case, the proposed schemes can be applied in time division multiple access (TDMA) manner, i.e., the sources deliver data one after the other. We will investigate extending the proposed schemes to the case of simultaneously delivering data over multiple source–destination pairs in the future.

## Figures and Tables

**Table 1:** Definitions of notations used.

Notation	Definition
*R_i_*	The set of the nodes on the *i*-th path
|*R_i_*|	The number of the elements in *R_i_*
(*i*, *j*)	The *j*-th node on the *i*-th path
*P_b_*_(*i*,*j*)_	Residual energy before data delivery for (*i*, *j*)
*P_a_*_(*i*,*j*)_	Residual energy after data delivery for (*i*, *j*)
*H*_(*i*,*j*)_	Incoming energy during the period of data delivery for (*i*, *j*)
*d*_(*i*,*j*)_	The distance between nodes (*i*, *j*) and (*i*, *j* + 1)
*S*	The source node
*u*	The minimum transmission unit
*B*	The energy storage capacity
$\varepsilon_{0}$	Energy consumption in digital coding, modulation, filtering, and spreading of the signal, etc.
$\varepsilon_{1}$	Energy consumption of the transmitter power amplifier
*γ*	Path loss exponent
*M*	The number of chromosomes in a population in GA

**Table 2:** Parameter values.

Notation	Definition	Value
*u*	The minimum transmission unit	one octet
*B*	The energy storage capacity	5 Joule
$\varepsilon_{0}$	Energy consumption in digital coding, modulation, filtering, and spreading of the signal, etc	50 nJ/bit [20]
$\varepsilon_{1}$	Energy consumption of the transmitter power amplifier	10 pJ/bit/m^2^ [20]
*γ*	Path loss exponent	2
*M*	The number of chromosomes in a population in GA	400
